# Aspergilloma Superimposed Infection on Lymphoid Interstitial Pneumonia

**DOI:** 10.1155/2020/3151036

**Published:** 2020-01-18

**Authors:** Daniel Tran, Rajagopalan Rengan, James Lee, Alan Lucerna, James Espinosa

**Affiliations:** ^1^Department of Internal Medicine, Rowan University SOM/Jefferson—Stratford, Stratford, NJ, USA; ^2^Department of Emergency Medicine, Rowan University SOM/Jefferson—Stratford, Stratford, NJ, USA

## Abstract

We describe a case of a 27-year-old female without any prior underlying immunodeficiency syndromes who presented with hemoptysis secondary to subacute invasive pulmonary aspergillosis and subsequently diagnosed with lymphoid interstitial pneumonia (LIP). CT chest demonstrated bilateral interstitial disease with patchy opacities and multiple large cysts and bullae. Diagnosis was confirmed histologically after surgical lung resection of the mycetoma containing cavitation. Therefore, LIP should be suspected in patients presenting with opportunistic infections in the setting of cystic lung disease.

## 1. Introduction

Lymphoid interstitial pneumonia (LIP) is a diffuse interstitial lung disease characterized by dense infiltrations by polyclonal B and T lymphocytes and plasma cells, with hyperplasia of the bronchus associated lymphoid tissues. LIP might rarely also present as a cystic disease. Patients are commonly females in their fifth decade and frequently associated with a wide variety of underlying systemic diseases, most notably Sjogren syndrome, HIV, and other immunodeficiencies [1, 2]. Disease is mainly restricted to the lung and so presenting symptoms commonly include cough, dyspnea, and pleuritic chest pain. The clinical course is highly variable with a 5-year mortality between 33% and 50% [3]. Primary cause of death is usually due to end-stage lung disease, leading to respiratory failure.

Here we describe a case of a young female, without any known systemic immunodeficiencies, presenting with hemoptysis secondary to aspergilloma, leading to a diagnosis of LIP.

## 2. Case Presentation

A 27-year-old female presented with hemoptysis for the past several months worsening over the last couple days. She denies fevers, chest pain, dyspnea, weight loss, arthralgia, xerostomia or keratoconjunctivitis sicca. She was recently diagnosed with *Mycobacterium abscessus* infection on sputum culture after presenting with fevers and shortness of breath about 5 months prior. Chest X-ray did not show obvious cavitary lesions at that time and no CT chest was performed. She was originally treated with Clarithromycin and Cefoxitin via port infusion. However, her new infectious disease physician switched her to Amikacin, Clarithromycin, and Imipenem-Cilastatin after consulting with the National Jewish Hospital. She denies a history of incarceration or recent IV drug abuse. She does however admit to snorting heroin three years ago but had since abstained. She has a 4 pack-year smoking history and quit 2 years ago. Physical exam reveals mild scattered expiratory wheezes throughout and grade 3 digital clubbing on upper extremities.

Vital signs on arrival were blood pressure of 106/97 mmHg, heart rate of 111 beats per minute, respiratory rate of 18 breaths per minute, and a temperature of 98.0°F with oxygen saturation of 97% in room air.

**Figure 1:**
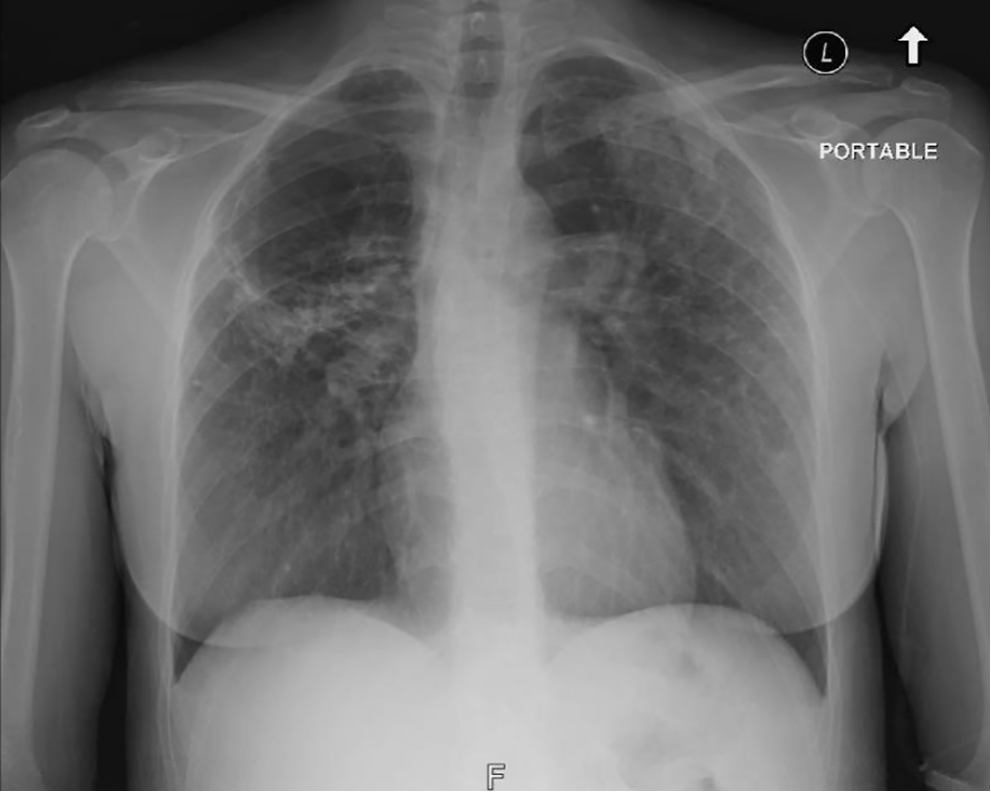
Chest X-ray demonstrating patchy interstitial opacities in bilateral upper lobes and a crescent sign in the left upper lobe.

**Figure 2:**
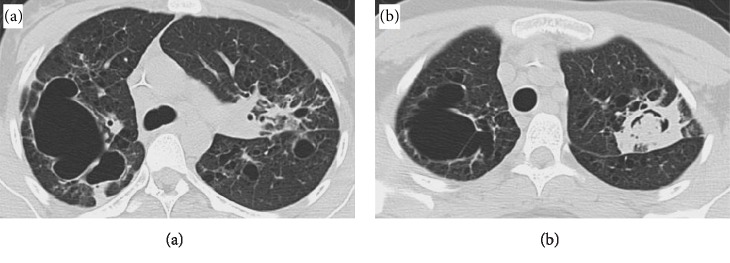
Computed tomography of multiple bilateral bullae and lung cavitations. (a) Largest bullae measuring up to 8 cm in the right upper lobe and filled with air. (b) Suspected mycetoma within the left upper lobe lung cavitation.

Chest X-ray demonstrated patchy interstitial opacities and a crescent sign in the left upper lobe (Figure 1). CT chest demonstrated bilateral interstitial disease with patchy opacities and multiple bullae and lung cavities. Largest cavity at the right upper lobe is air-filled, mostly thin walled and measures up to 8 cm (Figure 2(a)). 3 cm cavity at left apex is thick-walled and contains fluid density material suspicious for a mycetoma (Figure 2(b)). Mediastinal adenopathy was also present. CT abdomen pelvis was negative for any masses, hepatomegaly, splenomegaly, or other acute abnormalities.

Laboratory tests were negative for ANA, RF, SSA Sjogren Ab, SSB Sjogren Ab, A1AT, and VEGF. Total protein level was 7.3 g/dL and urine protein was negative. Serum protein electrophoresis was not performed. Interestingly, HIV was also negative. Bronchoscopy with bronchoalveolar lavage (BAL) was negative for malignant cells but showed many bronchial cells, broncho-alveolar macrophages and acute inflammation. Bronchial washings were positive for *Aspergillus*.

Due to her persistent hemoptysis and likely Aspergilloma, left lateral thoracotomy with left upper lobectomy and mediastinal lymph node dissections were performed. There were dense intrapleural adhesions throughout her chest cavity. Mediastinal lymph nodes were dissected from subaortic, para-aortic, and lung phrenic nerve.

**Figure 3:**
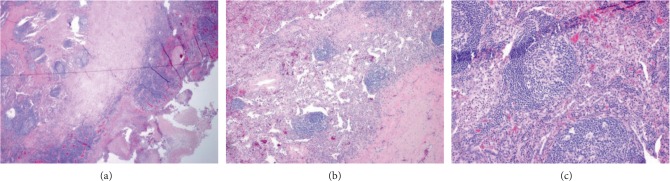
(a) low magnification view showing distribution of lesions. (b) extensive alveolar septal and interstitial infiltration of polyclonal lymphocytes, plasma cells and histiocytes in a peribronchovascular pattern with the presence of germinal centers. (c) magnified view of lymphocytic infiltration and lymphoid follicles.

Surgical resection of left upper lobe shows mycetoma with associated acute and chronic inflammation and reactive lymphoid infiltrate. Lymph nodes showed lymphoid infiltrate with reactive follicles in a peri-bronchiolar and alveolar distribution. Melan-A and HMB-45 were negative. CD1A and S-100 highlighted scattered positive cells. CD4 and CD8 highlighted a mix of CD4 and CD8 positive cells. EBV-ISH displayed scattered positive cells. There were no evidence of *Mycobacterium abscessus* infection in the resected lung specimen. Overall immunohistochemical stains and distribution of lymphoid infiltrates were consistent with lymphoid interstitial pneumonia (Figure 3). She was discharged on 20 mg of prednisone daily.

**Figure 4:**
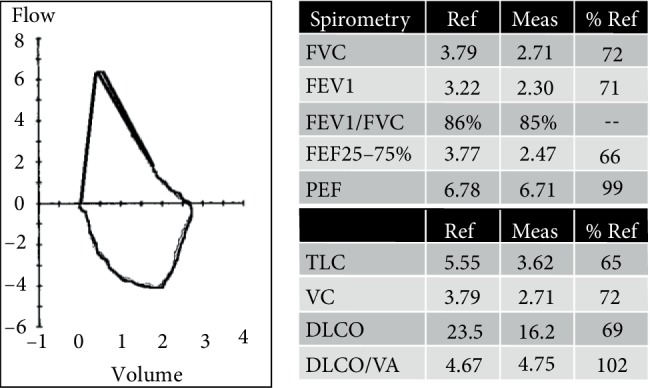
(Left) spirometry demonstrating a flow-volume loop with a restrictive flow pattern. (Right) spirometry measurements and diffusion capacity demonstrating reduced TLC and reduced DLCO consistent with interstitial lung disease.

Our patient was seen in the office 4 months after thoracic surgery. She completed pulmonary rehabilitation and is doing well, no longer having limitations in activity or further episodes of hemoptysis. Pulmonary function testing demonstrated a restrictive flow pattern and low total lung volumes, consistent with parenchymal disease and volume loss from lung resection (Figure 4). However, her total lung capacity (TLC) had improved by 7% from her baseline. Diffusing capacity of the lungs for carbon monoxide (DLCO) is depressed but still improved by 12%, also consistent with interstitial lung disease. She continues to abstain from smoking tobacco and snorting heroin. Daily corticosteroids were discontinued and additional immunosuppressive agents were not necessary at this time. She had completed her entire course of Amikacin, Clarithromycin, and Imipenem-Cilastatin, for a total of 9 months.

## 3. Discussion

Cystic lung disease comprises of four main subtypes: lymphangioleiomyomatosus (LAM), pulmonary Langerhans cell histiocytosis (PLCH), Birt-Hogg-Dube syndrome (BHD) and lymphoid interstitial pneumonia (LIP). Extrapulmonary manifestations and radiographic findings are essential in distinguishing between these diseases. Pulmonary function testing (PFT) shows restrictive physiology with reduced FVC and FEV1, elevated FEV1/FVC and reduced total lung capacity. Typical CT findings of thin-walled, predominantly mid-zoned perivascular cysts associated with ground-glass attenuation and centrilobular nodules in a nonsmoker is highly suggestive of LIP [1, 4]. Other frequent imaging findings include bilateral bronchovascular and interlobular septal wall thickening with lower lobe predilection. Furthermore, patients with associated underlying connective tissue, autoimmune or immunodeficiency disorder should raise the suspicion for LIP [5, 6].

Our patient presented with cough and digital clubbing but was relatively asymptomatic otherwise. She was tested negative for HIV, Sjogren antibodies, antinuclear antibodies and rheumatoid factor. Moreover, mediastinal lymphadenopathy which was concerning for an underlying malignant process was ruled out by immunohistochemistry results of the surgical biopsy and by CT pan-scan. LIP was only then confirmed histologically after concerns for subacute invasive pulmonary aspergillosis were addressed. Given the negative workup so far, a diagnosis of idiopathic LIP was considered. However, further studies to exclude EBV infection or dysgammaglobulinemia were not completed before our patient was unfortunately lost to follow up.

**Figure 5:**
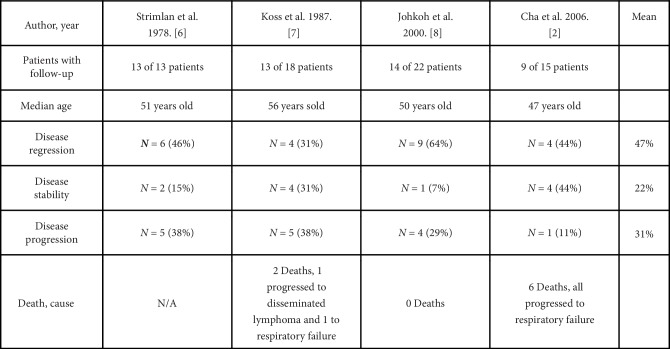
Summary table of follow-up data in four case series for lymphoid interstitial pneumonia showing disease regression, stability, and progression (see [2, 6–8]).

The natural disease progression and prognosis of LIP are poorly understood. LIP can regress and resolve spontaneously, stabilize without new cyst formation, or even progress to pulmonary fibrosis or lymphoma leading to death with median survival time ranging from 5 to 11.5 years despite treatment [3]. Follow-up data is limited, but four case series are summarized showing an average of 47% with improving disease, 22% with disease stability and 31% with disease progression (Figure 5). Despite the small population in these studies, this provides further insight that the natural course of LIP is variable and uncertain. Pulmonary aspergillosis may potentially be a missed comorbidity of LIP that could affect the natural course of the disease. Perhaps secondary infections are potential complications of LIP and an opportunistic infection with *M. abscessus* and *Aspergillus* was a herald for progression of our patient's disease.

There is currently no consensus on first-line therapy for LIP, as controlled clinical trials have not yet been reported. The latest therapeutic regime consists of corticosteroids alone versus immunoglobulin therapy modification or cytotoxic drugs [4, 8]. Further complicating matters include LIP in the presence of commonly associated immunodeficiency syndromes such as rheumatoid disease, HIV infections and dysgammaglobulinemia in which immunosuppressive agents may further exacerbate opportunistic infections. Due to the rarity of LIP, prognosis, and long-term complications are poorly understood. Our patient improved clinically and with partially recovered lung function as evident by pulmonary function testing after short-term corticosteroid therapy, which could enforce the notion for steroids alone as an initial treatment option.

## 4. Conclusion

In summary, we report a case of hemoptysis secondary to invasive pulmonary aspergillosis superimposed on lymphoid interstitial pneumonia without any known associated underlying immunodeficiency syndromes. Furthermore, pulmonary aspergillosis and may be a missed comorbidity associated with progression of LIP. Therefore, LIP should be suspected in patients presenting with opportunistic infections in the setting of cystic lung disease.
